# Mutations in epidermal growth factor receptor and K-ras in Chinese patients with colorectal cancer

**DOI:** 10.1186/1471-2350-11-34

**Published:** 2010-02-26

**Authors:** Zuo Yunxia, Cao Jun, Zhu Guanshan, Lu Yachao, Zhou Xueke, Li Jin

**Affiliations:** 1 Department of Medical Oncology, Fudan University Cancer Hospital, Shanghai Medical School, Shanghai 200032, PR China; 2 Innovation Center China, AstraZeneca Global R&D, 898 Halei Road, Shanghai 201203, PR China

## Abstract

**Background:**

Mutations of EGFR and K-ras are biomarkers for predicting the efficacy of targeting agents in non-small-cell lung cancer (NSCLC) and colorectal cancer (CRC). Data on the gene mutation status of EGFR and K-ras in Chinese patients with CRC are limited.

**Methods:**

EGFR mutations in exon 18-21 and K-ras mutations in exon 1 and 2 were detected in tumor samples from 101 Chinese patients with CRC by polymerase chain reaction-single strand conformational polymorphism. The relationship between patients' characteristics and survival time and gene mutation status were analyzed using the Statistical Package for the Social Sciences.

**Results:**

Only two samples (2.0%) had EGFR mutations in exon 18 or 21, and 33 of 101 samples (32.7%) had K-ras mutations in codon 12, 13, 45, 69, or 80. Univariate analysis suggested that differentiation might be correlated with K-ras mutations (*p *= 0.05), which was confirmed by a logistic regression model (*p *= 0.04). The median overall survival (OS) and median survival after metastasis were 44.0 and 18.0 months, respectively, in the mutant K-ras group, and 53.3 and 19.0 months, respectively, in the wild K-ras group. K-ras mutation was not an independent prognostic factor for OS or survival after metastasis (*p *= 0.79 and 0.78, respectively).

**Conclusions:**

In Chinese patients with CRC, EGFR mutations were rare, and K-ras mutations were similar to those of Europeans. New mutations in codons 45, 69, and 80 were found in the Chinese population. Poor differentiation was an independent factor related to K-ras mutations.

## Background

Epidermal growth factor receptor (EGFR) is highly expressed in many malignancies, including head and neck cancer, lung cancer, and colorectal cancer[1]. Upregulated EGFR is correlated with both poor prognosis and increased metastatic potential in numerous epithelial malignancies[2,3]. Further investigation has recently revealed that, in patients with non-small cell lung cancer (NSCLC) with mutated EGFR, higher response rates and longer survival time could be achieved with the use of the EGFR tyrosine kinase inhibitor gefitinib. The mutations were centered on exon 18-21 of the EGFR tyrosine kinase domain and were mostly detected in Asian patients with NSCLC, which suggested that gefitinib played an important role in the Chinese population[4,5]. It has been reported that the mutation incidence in colorectal cancer (CRC) was approximately 0.34% to 3.00% in western countries [6,7]. In contrast, the mutation incidence was reported to be as high as 12% in a study from Japan of 33 patients with CRC[8]. However, the differences between Western and Eastern patients with CRC have not been clearly documented, and no data from Chinese patients with CRC are currently available.

The K-ras gene is located downstream in the EGFR signal pathway. The Ras protein is activated transiently as a response to extracellular signals, such as growth factors, cytokines, and hormones that stimulate cell surface receptors. It can switch between an inactive state, in which the proteins are bound to guanosine-diphosphates, and an active state, in which conversion to guanosine-triphosphate (GTP) occurs. Mutant activated forms of Ras proteins have an impaired intrinsic GTPase activity, which renders the protein resistant to inactivation by regulatory GTPase-activating proteins[9]. Approximately 20% to 50% of patients with colorectal adenocarcinoma have a K-ras mutation, and 90% of the mutations were found in codons 12 and 13, followed by codon 61[10]. Studies have recently confirmed that a mutant K-ras gene could lead to resistance to cetuximab and panitumumab in metastatic CRC (mCRC), suggesting that K-ras status should be considered when selecting patients with mCRC as candidates for panitumumab or cetuximab monotherapy[11,12].

Mutations in both EGFR and K-ras will promote the progression of resistance to anti-EGFR targeting therapy. Limited data in the Chinese population prompted this study, which was performed to explore mutations in EGFR and K-ras gene in Chinese patients with CRC and provide evidence for the efficacy-prediction of EGFR targeting therapeutic strategies.

## Methods

### Tissue samples

Study approval was provided by the Medical Ethical Committees of the Fudan University Cancer Hospital, Shanghai, China, a specialist cancer hospital serving mainland China (60% of patients attend from other provinces, many of whom have late-stage disease). All samples of colorectal adenocarcinoma from operations performed at the Fudan University Cancer Hospital between January 2004 and March 2006, for which full information was available, were included. 101 samples that fit the inclusion criteria were obtained. The slides were first selected under the microscope to ensure that it contained sufficient tumor material. The paraffin-embedded tumor tissue blocks were then dissected into 8-10 μm sections for PCR sample preparation.

### DNA extraction

First, 200-μL cell lysis solution and 20-μl proteinase K stock solution were added to the tissue samples and incubated for 1 hour at 60°C, then for 20 minutes at 70°C. Subsequently, DNA was extracted after 72 hours at 37°C, protein was removed, and the DNA was precipitated using 100% 2-propanol and dissolved in hydration buffer.

### Polymerase chain reaction amplification and product purification

Four fragments of exon 18-21 of the EGFR gene and two fragments of exon 1 and 2, which included K-ras codons 12, 13, and 61, were amplified from isolated genomic DNA using polymerase chain reaction (PCR).

### Primer

Primers of the EGFR exon 18-21 were as follows:

First reaction of exon 18: 5' GAC CCT TGT CTC TGT GTT CTT GT 3', 5' CTT TGG TCT GTG AAT TGG TCT C 3';

Second reaction of exon 18: 5' TGA GGA TCT TGA AGG AAA CTG AAT 3', 5'TGC CA G GGA CCT TAC CTT ATA CA 3';

First reaction of exon 19: 5' CCC CAG CAA TAT CAG CCT TAG 3', 5' TGA AGT TTT AGG ATG TGG AGA TGA 3';

Second reaction of exon 19: 5'GTG CAT CGC TGG TAA CAT CCA C 3',5' CAG AGC AGC TGC CAG ACA TGA G 3';

First reaction of exon 20: 5' TCC ACA TCC TAA AAC TTC ACA GC 3', 5' GCA GAC CGC ATG TGA GGA TC 3'.

Second reaction of exon 20: 5' CCA TGA GTA CGT ATT TTG AAA CTC 3', 5' TTA TCT CCC CTC CCC GTA TC 3';

First reaction of exon 21: 5' CTA ACG TTC GCC AGC CAT AAG TCC 3', 5' GCT GCG AGC TCA CCC AGA ATG TCT GG 3'; and

Second reaction of exon 21: 5' CTG AAT TCG GAT GCA GAG CTT C 3', 5' GAG AGC ATC CTC CCC TGC ATG TG 3'.

Primers of the two fragments of K-ras exon 1 and 2 were as follows:

First reaction of exon 1: 5' TCT TAA GCG TCG ATG GAG GAG 3', 5' TGA CAT ACT CCC AAG GAA AGT AAA G 3';

Second reaction of exon 1: 5' ATA CAC GTC TGC AGT CAA CTG G 3', 5' CCT CTA TTG TTG GAT CAT ATT CGT 3';

First reaction of exon 2: 5' ATG GGT ATG TGG TAG CAT CTC AT 3', 5' AAG TTA CTC CAC TGC TCT AAT CCC 3';

Second reaction of exon 2: 5 'TTT TCC TGA CTA TTG ATG ATG TTG 3',

5' GCA TGG CAT TAG CAA AGA CTC 3'.

The PCR cycling variables are listed below:

EGFR exon 18

One cycle at 96°C for 5 minutes, 10 cycles each at 95°C for 10 seconds, 57°C for 45 seconds, and 72°C for 25 seconds (0.5°C descended at each cycle), 40 cycles each at 95°C for 20 seconds, 52°C for 30 seconds, and 72°C for 30 seconds, followed by one cycle at 72°C for 5 minutes.

EGFR exon 19 and exon 21

One cycle at 95°C for 5 minutes, 20 cycles each at 94°C for 20 seconds, 58°C for 45 seconds, and 72°C for 25 seconds (0.5°C descended at each cycle), 35 cycles each at 95°C for 20 seconds, 48°C for 30 seconds, and 72°C for 25 seconds, followed by one cycle at 72°C for 3 minutes.

EGFR exon 20

One cycle at 96°C for 5 minutes, 10 cycles each at 95°C for 10 seconds, 60°C for 30 seconds, and 72°C for 20 seconds (0.5°C descended at each cycle), 40 cycles each at 95°C for 20 seconds, 55°C for 30 seconds, and 72°C for 30 seconds, followed by one cycle at 72°C for 4 minutes.

K-ras exon 1 and exon 2

One cycle at 95°C for 5 minutes, 20 cycles each at 94°C for 20 seconds, 58°C for 45 seconds, and 72°C for 25 seconds (0.5°C descended at each cycle), 30 cycles each at 95°C for 20 seconds, 48°C for 30 seconds, and 72°C for 25 seconds, followed by one cycle at 72°C for 3 minutes.

The same cycling conditions were used for both external and internal primers.

The internal products were checked for purity and size by electrophoresis on a 2% agarose gel and subsequently used for direct sequencing.

### Mutation analysis

The resulting PCR products were sequenced using the Big Dye Terminator Cycle Sequence Ready Reaction Kit (Applied Biosystems, Foster City, CA, USA) following the manufacturer's protocol. The EGFR and K-ras sequences were aligned and analyzed with the Sequencing Analysis v5.1 software.

### Survival

Overall survival (OS) was defined from the day of operation to the day the patient died of the disease or the day follow-up ended (August 2008). Survival after metastasis was defined from the day metastasis was confirmed to the day the patient died of the disease or the day follow-up ended (August 2008). Follow-up was done by telephone.

### Statistical analysis

Differences in mean values were evaluated using *t*-test. Differences in categorical variables, such as sex, age, and tumor sub-localization, between patients with and without K-ras mutations were evaluated for significance with chi-squared test (or Fisher's exact test when necessary). Multivariate analysis was done with a logistic regression model. Kaplan-Meier method and log-rank tests were used for univariate survival analysis, and a Cox regression model was used in multivariate survival analysis. A *p *value of ≤0.05 was considered statistically significant. Statistical analyses were performed using the Statistical Package for the Social Sciences version 12.0.

## Results

### Patients' Characteristics

No significant differences in age were found between the 59 male patients and 42 female patients (57.3 years vs. 54.8 years, respectively, *p *= 0.331, *t*-test). Other characteristics, such as tumor sub-localization, pathology, differentiation, and International Union Against Cancer (UICC) stage, are listed in Table 1.

**Table 1:** Patients' characteristics.

	Number (%)
Number of patients	101 (100)
Sex	
Male	59 (58.4)
Female	42 (41.6)
Sub-localization	
Colon	54 (53.5)
Rectum	47 (46.5)
Adenocarcinoma	
Tubular/papillary	32 (31.7)
Mucinous	12 (11.9)
Others	57 (56.4)
Differentiation	
Good	2 (2.0)
Moderate	69 (68.3)
Poor	11 (10.9)
Unknown	19 (18.8)
Stage (UICC^a^)	
I	7 (6.9)
II	20 (19.8)
III	49 (48.5)
IV	25 (24.8)

^a^International Union Against Cancer.

### Epidermal growth factor receptor and K-ras gene mutation types

**Figure 1:**
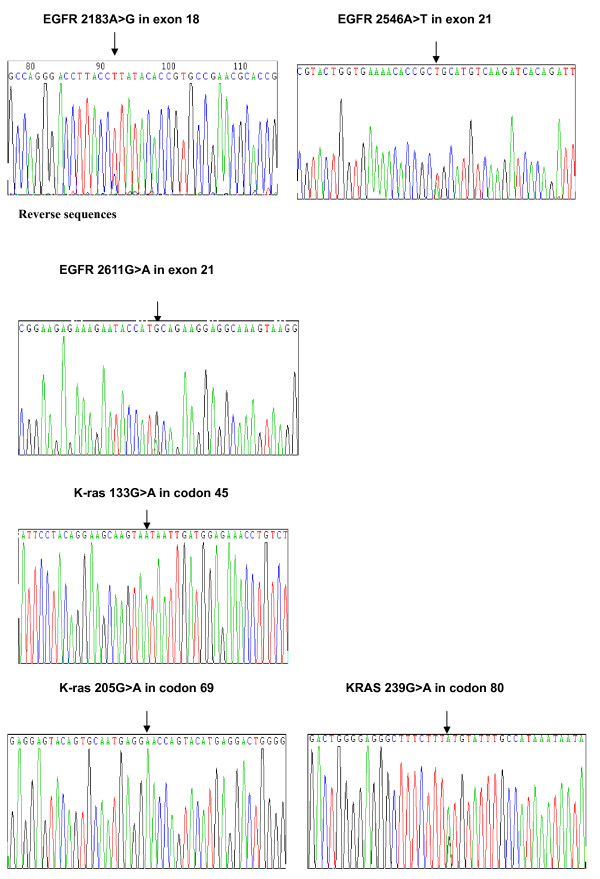
Epidermal growth factor receptor and K-ras mutations

Only 2 of 101 samples (2.0%) were found with mutations in exon 18 or 21 of the EGFR gene; both of which were substitutions and heterozygous and missense mutations. The mutation in one sample was 2183A>G in exon 18, leading to substitution of a glutamine by leucine acid (Gln849Leu). The other sample had two mutations of 2546A>T and 2611G>A both in exon 21, leading to transitions of Lys728Arg and Ala871Thr, respectively. No mutations were detected either in exons 19 or 20 (Table 2 and Fig 1).

**Table 2:** Epidermal growth factor receptor and K-ras mutations.

	Site	Wild type	Type of point mutation	Number of mutations (%)	Amino acid	Heterozygous/homozygous
EGFRa [SME1]	Exon 18	AAG	2183A>G	1 (33.3)	Gln849Leu	Heterozygous
	Exon 21	CAG	2546A>T	1 (33.3)	Lys728Arg	Heterozygous
	Exon 21	GCA	2611G>A	1 (33.3)	Ala871Thr	Heterozygous

K-ras	Codon 12	GGT	35G>A	16 (47.1)	Gly12Asp	Heterozygous
	Codon 12	GGT	34G>T	6 (17.6)	Gly12Cys	Heterozygous
	Codon 12	GGT	34G>A	3 (8.8)	Gly12Ser	Heterozygous
	Codon 12	GGT	34G>A	1 (2.9)	Gly12Ser	Homozygous
	Codon 12	GGT	34G>T	1 (2.9)	Gly12Cys	Homozygous
	Codon 12	GGT	35G>C	1 (2.9)	Gly12Ala	Heterozygous
	Codon 12	GGT	35G>A	1 (2.9)	Gly12Asp	Homozygous
	Codon 12	GGT	35G>T	1 (2.9)	Gly12Val	Heterozygous
	Codon 13	GGC	38G>A	1 (2.9)	Gly13Asp	Heterozygous
	Codon 45	GTA	133G>A	1 (2.9)	Val45Ile	Homozygous
	Codon 69	GAC	205G>A	1 (2.9)	Asp69Asn	Homozygous
	Codon 80	TGT	239G>A	1 (2.9)	Cys80Tyr	Heterozygous

^a ^Epidermal growth factor receptor.

In exon 1 and 2 of the K-ras gene, 34 mutations were found in 33 of 101 samples (32.7%); all of which were substitutions and missense mutations. Thirty of 34 mutations (88.2%) were found in codon 12. The most frequently observed mutations were heterozygous 35G>A transitions (16/34 mutations) and heterozygous 34 G>T transitions (6/34 mutations) in codon 12, leading to transitions of Gly12Asp and Gly12Cys, respectively. Other mutations in codon 12 included three heterozygous 34G>A (Gly12Ser), one homozygous 35G>A (Gly12Asp), one heterozygous 35G>C (Gly12Ala), one heterozygous 35G>T (Gly12Val), one homozygous 34G>T (Gly12Cys), and one homozygous 34G>A (Gly12Ser) transitions. One sample with heterozygous 35G>A transition in codon 12 was also found with homozygous 205G>A transition in codon 69, leading to a transition of Asp69Asn. The other mutations were one heterozygous 38 G>A (Gly13Asp) in codon 13, one homozygous 133G>A (Val45Ile) in codon 45, and one heterozygous 239G>A (Cys80Tyr) in codon 80 (Table 2 and Fig 1). No samples were detected with concurrent EGFR and K-ras gene mutations.

### Clinicopathological factors correlated with K-ras mutations

Differences in the categorical variables, including sex, age, tumor sub-localization, pathology, differentiation, and UICC stage, between patients with and without K-ras mutations were evaluated for significance with chi-squared test (Fisher's exact test when necessary). Only the factor of differentiation was found to be potentially correlated with K-ras mutations (*p *= 0.05), which showed that poorer differentiation might predict a greater possibility of K-ras mutations (Table 3), which was confirmed by multivariate analysis with a logistic regression model (*p *= 0.04).

**Table 3:** Patients' characteristics according to K-ras mutation status.

	K-ras mutation status		*p *Value
	**Mutation Number of patients**	**Wild type Number of patients**	
Number of patients	33	68	
Sex			0.16
Male	16	43	
Female	17	25	
Age (years)			0.89
≤56	17	34	
>56	16	34	
Sub-localization			0.26
Colon	15	39	
Rectum	18	29	
Adenocarcinoma			0.84
Tubular/papillary	10	22	
Others	23	46	
Differentiation			0.05
Good	2	0	
Moderate	18	51	
Poor	4	7	
Unknown	9	10	
Stage (UICC^a^)			0.18
I + II	6	21	
III + IV	27	47	

^a^International Union Against Cancer.

### Univariate survival analysis on K-ras status

**Figure 2:**
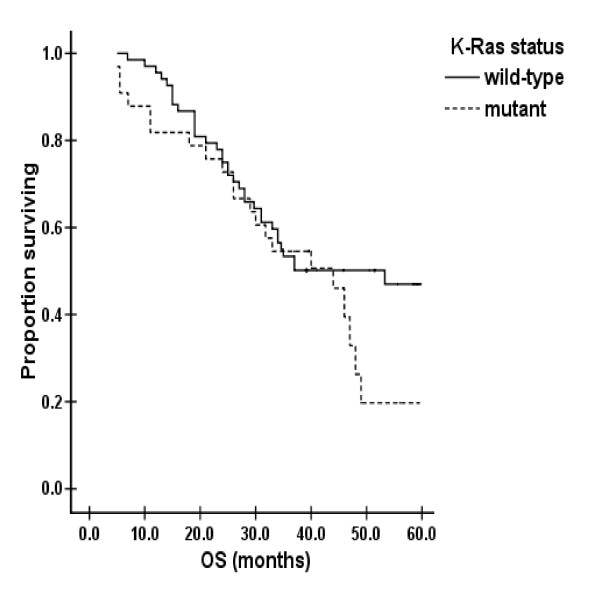
Overall survival and K-ras status

Median follow-up time of all the patients was 37.0 months. Median OS was 44.0 months (55 dead and 46 alive) in the whole group, 44.0 months in the K-ras mutation subgroup, and 53.3 months for the wild K-ras subgroup. No statistically significant difference was detected between the two subgroups (*p *= 0.23) [Fig 2].

**Figure 3:**
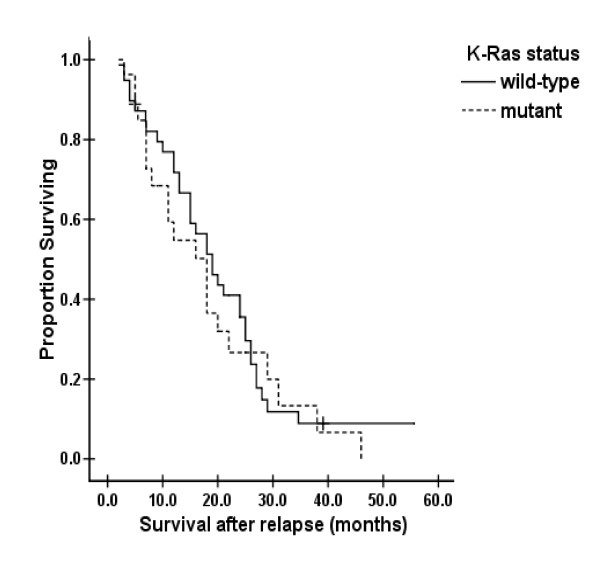
Survival after relapse versus K-ras status

Median survival after relapse of the 66 patients with confirmed metastasis was 18.0 months overall, 18.0 months in the K-ras mutation subgroup, and 19.0 months in the wild K-ras subgroup. No statistically significant difference was detected between the two subgroups (*p *= 0.59) [Fig 3].

### Multivariate survival analysis of K-ras status

Prognostic factors of sex, age, tumor sub-localization, pathology, differentiation, UICC stage, and K-ras status were analyzed using a Cox regression model. K-ras mutation was not an independent factor that correlated with OS or survival after metastasis (*p *= 0.79 and 0.78, respectively).

## Discussion

Better efficacy of the EGFR tyrosine kinase inhibitor (EGFR-TKI) gefitinib has been documented in Asian patients with NSCLC than in Caucasians[13]. The main reason for this finding is somatic mutations in the EGFR tyrosine kinase domain, which are more prevalent in Asian patients with NSCLC. Therefore, these mutations might be considered biomarkers for the use of gefitinib in the treatment of NSCLC. However, this study did not find similar results in Chinese patients with CRC. In total, 101 paraffin-section from colorectal adenocarcinoma samples were selected, and mutations in EGFR exon 18-21 were identified. Only 2.0% of the samples had mutations in exon 18 or 21, which was similar to that in the Western population. Substitutions of A2183G (Lys728Arg) in exon 18 and A2546T (Gln849Leu) and G2611A (Ala871Thr) in exon 21 were the only three mutations detected. No mutations were found in exon 19 or 20, which was different from the results in NSCLC, in which the principal mutation was a deletion in exon 19. The mutation incidence varied among different studies. Barber et al.[6] reported a 0.34% mutation incidence of Gly719Ser in 293 CRC samples in the USA, and Ogino et al.[7] found a 3.3% incidence of Gly724Ser in 30 samples, while no mutations were detected in Lynch et al.'s [4]study of 20 samples. However, a study from Japan reported by Nagahara et al.[8] found a relatively higher mutation incidence of 12% (4 of 33 samples). The mutations were all substitutions of Glu749Lys in exon 19 and Glu762Gly and Ala767Thr in exon 20. This study found that EGFR mutations were rare in Chinese patients with CRC, which was similar to the results from Western countries, but greatly different from those in Chinese patients with NSCLC.

EGFR-TKIs have shown satisfactory efficacy as first-, second-, or third-line therapy for patients with NSCLC with EGFR mutations. Similar studies of patients with CRC have been performed to investigate the application of EGFR-TKIs in this setting. A phase II study showed that two doses of gefitinib (250 mg/day and 500 mg/day) resulted in a partial response rate of 1% (1 of 110 patients) with a median progression-free survival of 1.9 months[14]. Townsley et al.[15] treated 39 patients with mCRC with erlotinib. Of 31 evaluable patients, 19 (61%) had progressive disease, and 12 (39%) had stable disease. However, there was a reduction in phosphorylated EGFR and phospho-extracellular signal-regulated kinase in tumor tissue after treatment. From these studies, gefitinib or erlotinib monotherapy seems unlikely to be effective for patients with CRC. The lower response rate for mCRC could be explained by a lower incidence of EGFR mutations than that found in NSCLC.

Further trials have focused on the combination of TKIs and chemotherapy. In a phase II study, the combination of capecitabine, oxaliplatin, and erlotinib seemed to have promising activity for patients who had received prior chemotherapy with a relatively high response rate (25%) and progression free survival (PFS) (5.4 months) compared with previous reports of either infusional 5-fluorouracil (5-FU), leucovorin, and oxaliplatin or capecitabine and oxaliplatin in a similar patient population[16]. Oxaliplatin, leucovorin, and 5-FU (FOLFOX-4) in combination with gefitinib in patients who failed irinotecan as a second-line therapy achieved a response rate of 33%, which was higher than that of FOLFOX-4 alone (10%)[17]. However, phase III trials are needed to eradicate selection bias before conclusions about the efficacy of the combination of EGFR-TKIs and chemotherapy can be confirmed.

In total, 34 mutations (32.7%) were found in 33 samples in this study, including common mutations in codons 12 and 13 and 3 novel mutations in codons 45, 69, and 80. The mutation incidence and most sites of mutation were all concordant with those reported in previous studies[18]. However, only one mutation in codon 13 was detected in the Chinese CRC samples, which is less than that reported in western studies. The three mutations found in codons 45, 69, and 80, which have not previously been reported, suggest that, occasionally, CRC in Chinese patients might have different biologic behavior and drug resistance although it is rarely detected. This is an exciting result because it suggests the possibility that other differences between Chinese and Western patients with CRC could be present, which could help in finding new individual treatments for Chinese patients. No mutations in codon 61 were detected in this study. It was reported in Western studies that approximately 90% of the activating mutations were found in codons 12 and 13 of exon 1 and ~5% in codon 61 located in exon 2[19-21]. Similarly, the mutation incidence of codon 61 was 0-4.8% in domestic reports. Therefore, our results were concordant with that of previous studies.

Differences in the categorical variables of sex, age, tumor sub-localization, pathology, differentiation, and UICC stage between patients with and without K-ras mutations were evaluated for significance with both chi-squared test and multivariate analyses. Only the factor of differentiation was potentially correlated with K-ras mutations (*p *= 0.05), which suggested that poor differentiation might predict K-ras mutations. This correlation was confirmed by multivariate analysis done with a logistic regression model. Duke's stage, lymph node metastasis, special pathology, and other factors were suggested as possible parameters related to K-ras mutations in previous studies, but no correlation has been achieved yet[22,23]. Currently, no conclusion can be made due to the lack of a meta-analysis, but this investigation will be continued with a larger sample.

Correlations between K-ras mutations and survival in CRC have been controversial. Although a notable shortening of OS was noted for the K-ras mutation subgroup compared with the wild K-ras subgroup, no statistical significance was detected by univariate and multivariate analysis. Similarly, no correlation was found between survival after metastasis and K-ras mutations by univariate and multivariate analyses. Some investigators have concluded that K-ras mutations could lead to poor survival, especially when anti-EGFR antibodies plus chemotherapy[11,24] were used, while others have found no significant correlation between K-ras mutations and survival[25,26], which was consistent with this study. However, some studies have shown that specific nucleotide mutations, inducing amino acid mutations, might be relevant to survival[18,27]. For example, Gly12Val mutations in codon 12 [27] and the G>A mutation in codon 13[18]both might induce poor survival. As only a few mutations in codon 12 and only one in codon 13 were detected in this study, and the follow-up time was insufficient, it was difficult to identify a definite correlation between K-ras mutations and survival. A further study with a larger sample size is ongoing, and more encouraging results might be found.

In NSCLC, K-ras mutations are related to the resistance of EGFR-TKIs, which are the opposite of EGFR mutations[28]. Some studies have recently indicated that K-ras mutations are responsible for the low response rate to EGFR monoclonal antibodies[11,29]. A similar result has been found in another large study, which confirmed that wild-type K-ras was required for panitumumab (an EGFR monoclonal antibody) efficacy in patients with mCRC [12]. At the 2009 American Society of Clinical Oncology Annual Meeting, there was an encouraging report of 540 samples from the CRYSTAL trial that were analyzed to evaluate treatment effect stratified by K-ras mutation status (Rougier et al. 2009, unpublished data). A statistically significant difference was seen in favor of cetuximab for best overall response (*p *= 0.0025), reducing the risk of progression (*p *= 0.0167) for patients with K-ras wild-type mutations, while no benefit was seen from the addition of cetuximab for the K-ras mutation subgroup. These data demonstrated that the treatment effect of cetuximab in patients with K-ras wild-type mutations was significantly enhanced compared with standard chemotherapy alone, whereas patients with a K-ras mutation could not be shown to benefit from cetuximab treatment. Therefore, K-ras status detection is recommended before the use of EGFR monoclonal antibodies. However, most patients with recurrent or metastatic disease in our study were not treated with EGFR monoclonal antibodies because they were mostly treated between 2004 and 2007. When cetuximab became available in 2006 in China, 13 patients were treated with this drug as a first- to third-line treatment, although many patients with recurrent or metastatic disease were not given cetuximab for financial reasons. Prior to 2006, conventional treatment was given. Due to the limited data on cetuximab treatment, we expect more studies on the relationship between K-ras mutations and cetuximab efficacy in the Chinese population because cetuximab is much more widely used in our hospital at present.

## Conclusions

EGFR mutations are rare in Chinese patients with CRC, which is similar to the results in those with NSCLC. Therefore, gefitinib might be ineffective for CRC. K-ras mutations were similar in Chinese patients with CRC to that in Western populations, and substitutions in codon 12 are the most common mutations. Poor differentiation is an independent factor related to K-ras mutation. K-ras mutation might not be a prognostic factor but may be a predictor of resistance to EGFR monoclonal antibodies.

## Competing interests

The authors declare that they have no competing interests.

## Authors' contributions

ZYX and CJ both participated in the design of the study, sample collections and follow-up of the patients, performed the statistical analysis, and drafted the manuscript. ZGS and LYC carried out the whole process of mutation detection. ZXK carried out sample selection and dissection. LJ conceived of the study and participated in its design, revision, and coordination. All authors read and approved the final manuscript.

## Pre-publication history

The pre-publication history for this paper can be accessed here:

http://www.biomedcentral.com/1471-2350/11/34/prepub
