# Mutations in epidermal growth factor receptor and K-ras in Chinese patients with colorectal cancer

**DOI:** 10.1186/1471-2350-11-95

**Published:** 2010-06-14

**Authors:** Zuo Yunxia, Cao Jun, Zhu Guanshan, Lu Yachao, Zhou Xueke, Li Jin

**Affiliations:** 1 Department of Medical Oncology, Fudan University Cancer Hospital, Shanghai Medical School, Shanghai 200032, China; 2 Innovation Center China, AstraZeneca Global R&D, 898 Halei Road, Shanghai 201203, China

## Correction

The authors would like to apologize for failing to attribute text in their manuscript [1]. The following sentences were not referenced correctly as a direct quotation:

"Ras protein is activated transiently as a response to extracellular signals, such as growth factors, cytokines, and hormones that stimulate cell surface receptors. It can switch between an inactive state, in which the proteins are bound to guanosine-diphosphates, and an active state, in which conversion to guanosine- triphosphate (GTP) occurs. Mutant activated forms of Ras proteins have an impaired intrinsic GTPase activity, which renders the protein resistant to inactivation by regulatory GTPase-activating proteins" [2].

In addition, the following errors were made in the Abstract, paragraph 2 of the Results and Table two of the original publication [1]:

1. The methods described in the Abstract as "polymerase chain reaction-single strand conformational polymorphism" should be "polymerase chain reaction and Sanger sequencing".

2.The sentence in the Results (paragraph 2) "leading to substitution of a glutamine by leucine acid (Gln849Leu)" should be replaced by "leading to substitution of a Lysine by Arginine (Lys728Arg)." The sentence "leading to transitions of Lys728Arg and Ala871Thr." should be "leading to transitions of Gln849Leu and Ala871Thr."

3.In the EGFR column of Table two of the original publication, Gln849Leu in the first row should be replaced by Lys728Arg. In the second row of the same column, Lys728Arg should be replaced by Gln849Leu. The corrections are shown in Table 1.

**Table 1:** Corrections to Table two "Epidermal growth factor receptor and K-ras mutations"

	Site	Wild type	Type of point mutation	Number of mutations (%)	Amino acid	Heterozygous/homozygous
EGFRa	Exon 18	AAG	2183A>G	1 (33.3)	Lys728Arg	Heterozygous
	Exon 21	CAG	2546A>T	1 (33.3)	Gln849Leu	Heterozygous
	Exon 21	GCA	2611G>A	1 (33.3)	Ala871Thr	Heterozygous

K-ras	Codon 12	GGT	35G>A	16 (47.1)	Gly12Asp	Heterozygous
	Codon 12	GGT	34G>T	6 (17.6)	Gly12Cys	Heterozygous
	Codon 12	GGT	34G>A	3 (8.8)	Gly12Ser	Heterozygous
	Codon 12	GGT	34G>A	1 (2.9)	Gly12Ser	Homozygous
	Codon 12	GGT	34G>T	1 (2.9)	Gly12Cys	Homozygous
	Codon 12	GGT	35G>C	1 (2.9)	Gly12Ala	Heterozygous
	Codon 12	GGT	35G>A	1 (2.9)	Gly12Asp	Homozygous
	Codon 12	GGT	35G>T	1 (2.9)	Gly12Val	Heterozygous
	Codon 13	GGC	38G>A	1 (2.9)	Gly13Asp	Heterozygous
	Codon 45	GTA	133G>A	1 (2.9)	Val45Ile	Homozygous
	Codon 69	GAC	205G>A	1 (2.9)	Asp69Asn	Homozygous
	Codon 80	TGT	239G>A	1 (2.9)	Cys80Tyr	Heterozygous

Corrections to Table 2 "Epidermal growth factor receptor and K-ras mutations" in the original publication [1] are shown in Table 1 of this Correction as follows: In the EGFR column of Table 2, Gln849Leu in the first row should be replaced by Lys728Arg. In the second row of the EGFR column, Lys728Arg should be replaced by Gln849Leu, as shown in Table 1 of this Correction.

## Pre-publication history

The pre-publication history for this paper can be accessed here:

http://www.biomedcentral.com/1471-2350/11/95/prepub
